# Does the heritability of cognitive abilities vary as a function of parental education? Evidence from a German twin sample

**DOI:** 10.1371/journal.pone.0196597

**Published:** 2018-05-08

**Authors:** Marion Spengler, Juliana Gottschling, Elisabeth Hahn, Elliot M. Tucker-Drob, Claudia Harzer, Frank M. Spinath

**Affiliations:** 1 Hector Research Institute of Education Sciences and Psychology, University of Tuebingen, Tuebingen, Germany; 2 Department of Psychology, Saarland University, Saarbruecken, Germany; 3 Department of Psychology and Population Research Center, University of Texas at Austin, Austin, Texas, United States of America; 4 Department of Psychology, Technical University Darmstadt, Darmstadt, Germany; Tilburg University, NETHERLANDS

## Abstract

A well-known hypothesis in the behavioral genetic literature predicts that the heritability of cognitive abilities is higher in the presence of higher socioeconomic contexts. However, studies suggest that the effect of socioeconomic status (SES) on the heritability of cognitive ability may not be universal, as it has mostly been demonstrated in the United States, but not in other Western nations. In the present study we tested whether the importance of genetic and environmental effects on cognitive abilities varies as a function of parental education in a German twin sample. Cognitive ability scores (general, verbal, and nonverbal) were obtained on 531 German twin pairs (192 monozygotic, 339 dizygotic, ranging from 7 to 14 years of age; *M*_age_ = 10.25, *SD* = 1.83). Data on parental education were available from mothers and fathers. Results for general cognitive ability and nonverbal ability indicated no significant gene x parental education interaction effect. For verbal ability, a significant nonshared environment (E) x parental education interaction was found in the direction of greater nonshared environmental influences on verbal abilities among children raised by more educated parents.

## Introduction

It is a well-known finding that cognitive abilities are heritable: depending on the age of the sample, the estimation method used, and the type of cognitive ability assessed, genetic differences between individuals account for between approximately 20% and 70% of the variance in cognitive abilities [1,2]. These omnibus heritability estimates, however, mask systematic transactions between genetic and environmental factors. Two forms of such a *gene-environment interplay* are gene-environment correlation (rGE)–in quantitative behavioral genetics the statistical phenomenon of nonrandom exposure to environments based on differences in genotype [3], and gene-environment interaction (G×E). G×E refers to the genetic sensitivity or susceptibility to environments [4]. It is important to note that G×E is usually limited to statistical interactions, i.e., the effect of genes depends on the environment and/or the effect of the environment depends on the genotype [4,5]. As a result, the magnitude of genetic contribution to the variance in a given phenotype can vary across levels of a measured environmental variable [6]. When we refer to G×E, we are referring to this statistical sense of interactions.

Age is one well replicated variable upon which the heritability (i.e., the proportion of phenotypic variance attributable to genetic variance; [4]) of cognitive ability depends. The general trend is that the magnitude of genetic variance increases gradually from early childhood to adulthood while shared-environmental variance decreases (e.g., [2,7]). Also, social context variables have been assumed to moderate the relative importance of genetic effects. Shanahan and Hofer suggested four processes by which social contexts may operate on the heritability of phenotypic traits [8]. The social context may *trigger a genetic diathesis* through the presence of a stressor, *compensate for a genetic diathesis* through the provision of enriched settings, or function as a *social control* mechanism that are placed on people to limit their behavior and choices (see [8]). One would therefore expect higher levels of genetic variance within the context of a more stressful, high risk, or less socially controlled environment in each of these instances [8–10]. Finally, a particular social context may operate as *enhancement*, encouraging the actualization of genetic potential for positive functioning [8]. As a result, the effect of genetic differences on a phenotypic trait should be more pronounced in enriched environments. Two theoretical perspectives, that of Bronfenbrenner and Ceci and that of Sandra Scarr, would both predict such a pattern of amplifying genetic differences with improving environments, but assume differing underlying processes that could lead to such a pattern at a given time. Bronfenbrenner and Ceci’s bioecological model emphasizes that proximal processes–which are defined as enduring forms of social interactions (e.g., the quality of interactions between child and caretakers)–facilitate the actualization of genetic potential [11] not only directly regarding cognitive processes, but also with respect to supportive personality characteristics such as Conscientiousness or self-regulation. Therefore, with improving proximal processes that are assumed to be part of enriched environments, underlying genetic differences can be more fully expressed and gain in importance. Scarr’s theoretical perspective emphasizes transactional processes between the individual and the environment as drivers of development (i.e., rGE; [12]). According to this assumption, children not only react but also select and evoke their environmental experiences based on their genetically influenced dispositions [2,13]. The heritability of a trait is assumed to be higher in enriched environments, as a result of presumably more opportunities to match one's genotype in these settings. In other words, what we statistically observe as a gene × social context interaction at a specific time may (partly) be the result of accumulated transactional processes over time. However, it should also be noted that, in most cases, it is not possible to disentangle these two proposed underlying mechanisms given the complex processes over time. Given that GxE and rGE probably operate simultaneously and interact over time, it might not be crucial whether it is rather the one or the other mechanism, but to take into account the interplay of enriched environments and the underlying genetic and environmental influences on cognitive ability, and to identify meaningful factors of this interplay.

Studies dealing with the question of whether the heritability of cognitive abilities might vary as a function of social context variables most often investigated the role of family socioeconomic status (SES). High SES homes are characterized by high levels of material resources, human, and social capital [14], and are assumed to provide better proximal processes (see e.g., [15,16]). Scarr was the first to report higher genetic variance in cognitive abilities in more privileged socioeconomic contexts in a sample of twins from the Philadelphia school system [17], a finding that was replicated in Sweden by Fischbein [18], and in the U.S. by Rowe et al. [19] (which is why the effect is commonly known as the Scarr-Rowe interaction; [20]). More recent empirical findings suggest that parental education (PE) and family income moderate genetic and environmental effects on cognitive abilities in the direction of higher heritability estimates in enriched socioeconomic contexts in (early) childhood [21,22], adolescence [3,6,23], and adulthood [24]. This pattern of result was widely interpreted as a confirmation of the bioecological model.

However, the empirical body of research on this question has not yielded a coherent set of results. A recent meta-analysis investigated 14 studies on G×SES interaction on objective measures of intelligence and academic achievement from different cultural backgrounds [25]. The authors reported a substantial heterogeneity of interaction effects: The G×E effect in the direction of a greater genetic variance at the higher end of the SES distribution was significant for U.S. samples, but was zero or even negative in European and Australian samples [25]. Yet, a recent study based on a sample of Norwegian conscripts reported a meaningful G×PE interaction on general cognitive ability [3]. While these differing findings among studies could potentially be attributable to differences in the age of the participants, or the operationalization of cognitive ability and SES, Tucker-Drob and Bates were unable to find meta-analytic evidence for such a moderation [25]. It is also possible that the inconsistent pattern of results is a cultural effect, for instance, smaller socioeconomic differences influencing cognitive ability in Europe compared to the U.S. [26], or between-nations variability in the effects of family SES on cognitive development [25]. Another important explanatory factor might be traced back to differing educational systems, differing school provisions, or differing access to education between nations. This, in turn could affect the G×SES interplay and its effect on cognitive ability.

Underexplored in extant research is the question whether G×SES effects might differ by the type of cognitive ability under study. Several studies have indicated that children living in lower-SES homes are more likely to have fewer educational resources, hence, tend to receive less verbal stimulation (e.g., [27–29]). This circumstance might be more closely linked to verbal test performance than to nonverbal test performance. One of the earlier studies on G×SES interaction on cognitive abilities by Rowe and colleagues reported different degrees of explained variance in a vocabulary test by genetic and environmental effects depending on maternal level of education: genes explained a large portion of the variance in families with well-educated mothers (74%), whereas in families with poorly educated mothers, genes only explained 26% of the variance [19]. Turkheimer and colleagues also reported results for verbal and nonverbal abilities separately. Although not statistically significant, the moderation for verbal IQ was in the same direction as for performance IQ, indicating higher heritability of cognitive abilities at higher levels of SES [22]. Based on data from over 4,000 pairs of four-year old British twins, Asbury and colleagues found no interaction for nonverbal ability. Yet, for verbal ability, significant interactions with measures of family chaos, and instructive and informal parent-child communication were found in the direction of higher heritability at the lower end of the environmental variable [30]. Note that the pattern of G×E interaction in this study was in the opposite direction than in the previously described studies. Finally, a recent study based on a large sample of adolescent Australian twins found no indication of significant genetic or environmental interactions, neither for verbal nor nonverbal IQ [31].

To summarize, although socioeconomic conditions are often considered to moderate the realization of genetic potential for cognitive ability, the pattern of results has not yielded a coherent picture, also with regard to different cognitive abilities. A recent meta-analysis [25] and a narrative report [32] indicated that the proposed G×SES interaction appears to occur reliably in the U.S., but not in other western societies [3]. In the present study we seek to extend the existing literature by a study investigating G×PE interaction in a German twin sample. As certain abilities may be differentially affected by socioeconomic contexts, we test separate models for verbal and nonverbal abilities in addition to a general factor of cognitive abilities.

## Method

### Participants

The sampling frame for the present study was the German twin study on Cognitive Ability, Self-Perceived Motivation, and School Achievement (CoSMoS; [33]) combined with data from a pilot study (*n* = 50 pairs) that was conducted to validate measures used in CoSMoS. Twin families were recruited through individual inquiries at registration offices in two German federal states (Thuringia and North Rhine-Westphalia; for details of the recruitment procedure see [34]. All families provided informed consent prior to their participation. A set of questionnaires including the cognitive test battery, zygosity information, and information on the educational background of the parents were mailed to the families.

The final sample for the present investigation consisted of 531 monozygotic (MZ) and dizygotic (DZ) child twin pairs with complete zygosity information (total *N* = 1,062), including data from 192 MZ (106 male, and 86 female pairs) and 339 DZ pairs (85 male, 103 female, 151 opposite-sex pairs). The twins ranged in age from 7 to 14 years (*M* = 10.25 years, *SD* = 1.83). Data on parental education (i.e., the highest educational degree held by the mother and father) were available from 526 mothers (age: 26–53 years; *M* = 40.17, *SD* = 4.30) and 517 fathers (age: 29–65 years; *M* = 42.65, *SD* = 5.17). All possible educational degrees were captured in the sample and approximately mirrored the respective distribution in Germany by the time the study took place [35]: no qualification, 0.4% of the mothers and 1.7% of the fathers; junior high school degree (German ‘Hauptschule’), 18.3% of the mothers and 26.9% of the fathers; second school certificate (German ‘Realschule’), 45.4% of the mothers and 27.3% of the fathers; high school diploma, 16.4% of the mothers and 13.7% of the fathers; university degree, 18.5% of the mothers and 26.4% of the fathers. Solely the percentage of individuals without any educational degree was lower compared to the German population.

### Measures

#### Zygosity determination

Zygosity was determined by the primary caregiver of the twins via a questionnaire assessing physical twin similarity in childhood (e.g., eye color, hair structure, time of dentition, etc.) and the frequency of twin confusion by significant others [36]. Zygosity assignment based on physical similarity questionnaires is a frequently used method for determining zygosity, because of their high accuracy rates of around 95% compared to DNA genotyping [37], their low costs, and their ease of use in large samples.

#### Parental education

Highest completed level of education of mothers and fathers was used as a proxy of SES. Parental education was classified on a 5-point ordinal scale from *“no qualification”* to *“university degree”* (1 = no qualification, 2 = junior high school degree, 3 = second school certificate, 4 = high school diploma, 5 = university degree). Parental education was calculated as an unweighted mean-score of maternal and paternal education (median = 3.00, variance = 0.93). The values were standardized and controlled for the average age of mother and father for the subsequent analyses. Parental education was normally distributed in the present sample (skewness = 0.30, kurtosis = -0.99).

#### Cognitive ability

General cognitive ability *(g)* was assessed by means of two verbal, and two nonverbal tests adapted from the German version of the Wechsler Intelligence Scale for Children [38], and the German Cognitive Ability Test [39]. The two nonverbal tests included the subscales “Figural Classification” and “Figural Reasoning” from the KFT 4–12+R (25 items each). The two verbal scales consisted of the subtest “Vocabulary” from the KFT 4–12+R (25 items) and the subtest “General Knowledge” of the WISC-III (depending on age between 18 and 21 items). The subtests of the KFT 4–12+R were administered to every age group as described in the manual (see [39]). The reliability and validity of both instruments are well established in German subsamples of children with different educational background and across different age groups.

Since the families in the CoSMoS sample lived in different regions of Germany, personal testing by the research team was not feasible. Therefore, particular attention was placed on the selection and usability of a cognitive test-battery suitable for parent-administration (49.7% of the sample) or administration by a trained interviewer over the phone (50.3%). In the case of parental home testing, parents were provided with detailed instructions on how to administer the cognitive testing. After completion, parents returned the test booklets to the CoSMoS office for scoring. Both methods have been successfully used in the population-based Twins Early Development Study (TEDS; [40]) with results indicating that both methods offer a reliable, valid, and time-saving alternative compared to face-to-face testing [40]. In addition, we tested both methods separately in non-twin samples in Germany in comparison to face-to-face testing with satisfying comparability between the methods. The equivalence of both types of administration for the present sample was confirmed by means of strong measurement invariance (χ^2^(*df* = 9, *p* = .28) = 10.89; CFI = .99; RMSEA = .02; LRT: χ^2^(*df* = 7, *p* = .25) = 9.01). For the purpose of the present investigation we calculated three separate cognitive ability scores as standardized sum scores of correct answers: a verbal cognitive ability score, a nonverbal cognitive ability score, and a total cognitive ability score (i.e., the sum of correct answers of all four subtests).

### Data analyses

#### The basic twin model

Behavior genetic analyses are based on the comparison of the trait similarity between MZ twins, who are genetically identical, and DZ twins, who share on average 50% of their segregating genes. The observed variance of a phenotype is most often decomposed into additive genetic (A; the sum of all genetic influences), shared environmental (C; common environmental influences for twins), and nonshared environmental (E; individual-specific environmental influences, also includes measurement error) variance components. The assumptions of the classical twin model, and their validity, have been discussed in detail elsewhere [41,42].

The OpenMx package for R [43] on raw data was used to fit structural equation models with full-information maximum likelihood estimation to the phenotypic covariance between twins. The relative fit of a model is evaluated by minus two times the log likelihood statistic (-2LL). The Akaike`s Information Criterion (AIC; [44]), the Bayesian information criterion (BIC; [45]), and the chi-square value were used for a general evaluation of the model fit.

#### Gene-environment interaction model

The classic twin model assumes uniform genetic and environmental influences over the range of a given trait or ability. This results in model estimates reflecting average, population-level genetic and environmental influences, while masking systematic transactions between specific genetic and environmental factors. Hence, in addition to the main effect of genes and environment (labeled “main effects model” in the following), multiple interactive effects of genes and environment can play an important role.

**Fig 1:**
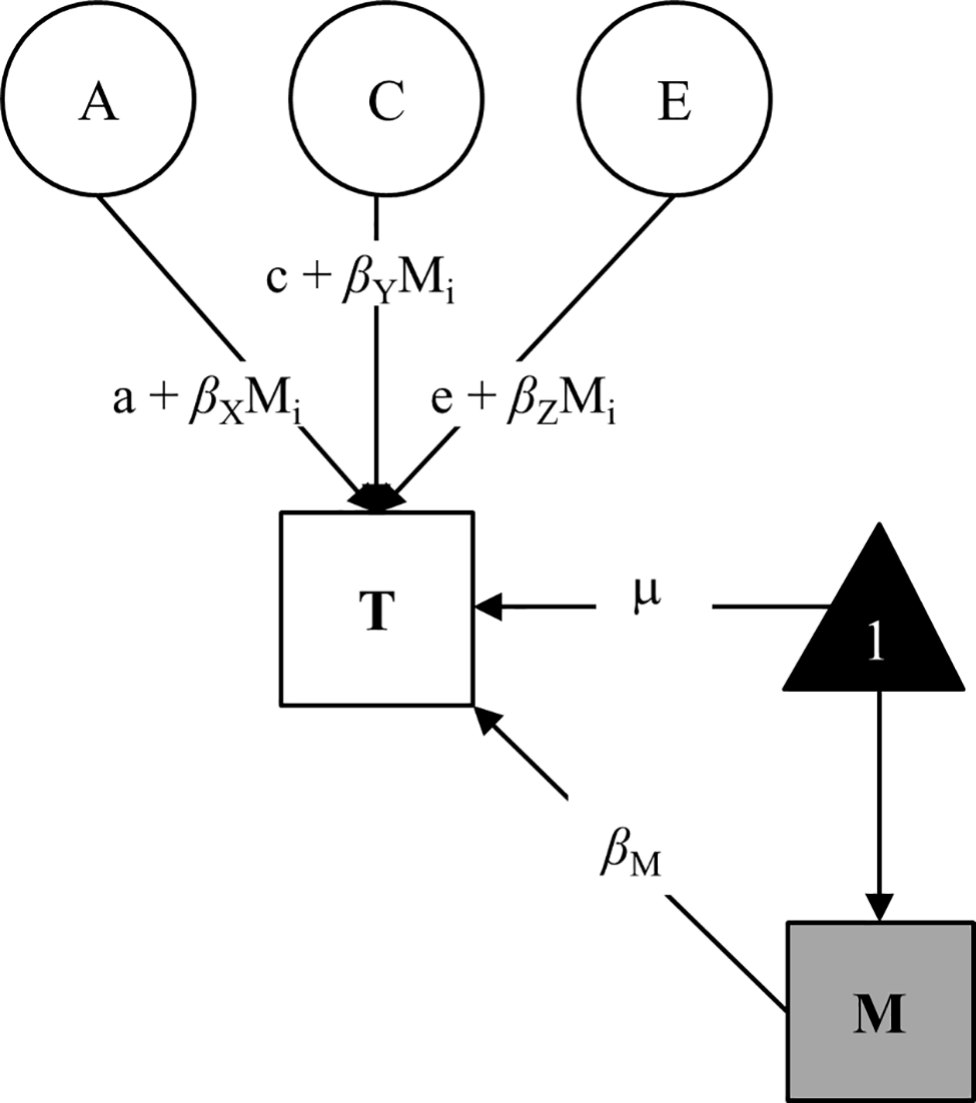
Continuous moderator model for a single twin (see [47]). The measured moderator (M) has a mediating or main effect (*β_M_*) on the trait (T), as well as a potential moderating effect on the variance components of the residual (after the main effect has been partialled out). A, C, and E represent additive genetic, shared environmental, and nonshared environmental influences on the trait; a, c, e, are the unmoderated elements of genetic, shared, and nonshared path coefficients; M_i_ is the measured moderator level for the i_th_ twin pair (both twins in a pair have the same value for obligatorily-shared moderators like SES); μ = the mean of the trait (T); 1 = the constant by which μ is multiplied, values of the trait are given by 1μ+ *β_M_*.

Fig 1 displays a classic twin model that has been expanded to include a moderation component. This continuous moderator model [46] allows to test whether genetic and environmental effects found within the classic model change as a linear function of the moderator, after accounting for the main effect of the moderator variable on the outcome (i.e., the effect of PE on mean levels of cognitive ability). That is, the moderator variable is allowed to have a main effect on the trait, as well as a moderating effect on the residual A, C, and E components of the trait. The phenotypic variance (P) of the trait T in the specified interaction model is decomposed as P_T_ = (*a* + *β_x_M_i_*)^2^ + (*c* + *β_y_M_i_*)^2^ + (*e* + *β_z_M_i_*)^2^. Thus, in the moderation model, the additive genetic coefficient is a linear function of the moderator M, i.e., *a* + *β_x_M_i_*, where *β_x_* is the unknown parameter to be estimated, *M_i_* represents the family-wide moderator value of the i_th_ twin pair, and *a* gives the average unmoderated additive genetic effect on the trait. The significance of the moderating effect of PE is indicated by a significant *β_x_*, *β_y_* or *β_z_* coefficient. The pathway *μ* + *β_M_* models the main effects of the moderator on the outcome. The *main* effect of the continuously measured environmental variable on T is assessed by estimating the value of *β_M_*. Also included in this pathway are potential rGE effects between M and T. Purcell demonstrated that inclusion of the main effect of the measured environment prevents bias in the estimation of G×E interactions resulting from unspecified rGE [46]. Thus, any unmeasured covariation between PE and cognitive ability is included in the means model and is therefore partialled out in the continuous moderator model [46]. It should also be noted that the model does not allow an explicit examination of rGE if the moderator is the same for both twins (e.g., family SES) because such analyses require within-twin pair variation.

To investigate the moderating effect of PE on the genetic architecture of different components of cognitive ability, the classic continuous moderation model was applied to the sample using (1) the total cognitive ability score, (2) the verbal component and (3) the nonverbal component. Model fitting included the estimation of eight parameters in the full moderation model: the classical a, c, e components, the moderation components *β_x_*, *β_y_*, *β_z_*, the main effect of the moderator *β_M_* (M), and the mean of the trait. Dropping all interaction parameters results in a main-effects-only model. We report the estimates for the full moderation models and the main-effects-only models and follow the general recommendation by reporting the unstandardized ACE estimates [46].

In addition to the classic continuous moderator model, which allows for linear moderation of the regression effect of ACE on the phenotype, we adopted a recently developed non-parametric approach to gauge the shape of any interactions detected at statistically significant levels by the classic Purcell model. Local structural equation modeling (LOSEM; see [48,49]) provides a non-parametric estimate of moderated trends by estimating multiple structural equation models that differ with respect to the assigned focal value of the moderator that is used to calculate the weights.

## Results

### Descriptive statistics

Means, standard deviations, skewness, and intra-class correlations (ICC) of the three cognitive ability scores (i.e., total score, verbal, and nonverbal) are presented in Table 1. There was no indication of significant differences between zygosity or sex groups with respect to means, standard deviation and skewness. The skewness statistics indicated that the cognitive ability scores were approximately normally distributed. ICCs were used to preliminary gauge the relative impact of genetic and environmental effects on cognitive abilities. Doubling the difference between MZ and DZ twins provides a rough estimate of the heritability of the trait, ranging from 28% - 32%) in the present sample.

**Table 1:** Descriptive statistics by zygosity for cognitive abilities.

		Cognitive Abilities		
		Total	Verbal	Nonverbal
Mean (SD)				
	Full sample	62.50 (15.25)	27.53 (7.47)	34.97 (10.01)
	MZ	62.62 (15.12)	27.18 (7.74)	35.44 (9.67)
	DZ	62.43 (15.34)	27.73 (7.31)	34.70 (10.20)
	Male	62.79 (15.01)	27.83 (7.32)	34.95 (9.83)
	Female	62.20 (15.50)	27.23 (7.60)	34.98 (10.19)
Skewness				
	Full sample	-0.56	-0.39	-0.72
	MZ	-0.55	-0.41	-0.74
	DZ	-0.56	-0.37	-0.71
ICC [95% CI]				
	MZ	.81 [.75; .85]	.78 [.72; .83]	.72 [.64; .78]
	DZ	.66 [.60; .72]	.63 [.56; .69]	.55 [.47; .62]

*N* = 1,062 individuals; SD = standard deviation; MZ = monozygotic twins; DZ = dizygotic twins; ICC = intra-class-correlation; CI = confidence interval.

Table 2 lists the phenotypic correlations between the relevant variables. PE as our proxy for family SES showed a moderate relation (between *r* = .19 and .23) with the cognitive ability subtests. Raw scores of the verbal and nonverbal scales were residualized for the effects of age and sex before conducting twin analyses to avoid inflated twin similarities and family-differences [50].

**Table 2:** Bivariate phenotypic correlations between parental education and cognitive abilities.

	Cognitive abilities						
Variables	GK	V	FC	FR	Total	VB	Non-VB
Parental education (PE)	.21	.19	.18	.17	.23	.22	.19
General knowledge (GK)		.62	.49	.39	.74	.87	.48
Vocabulary (V)			.45	.41	.76	.93	.48
Figural classification (FC)				.60	.82	.52	.87
Figural reasoning (FR)					.81	.44	.92
Total Score						.83	.91
Verbal (VB)							.53

Correlations are based on one randomly selected member of each twin pair (*n* = 531). All correlations are significant at *p* < .001.

### Main effects models

Prior to analyses, the assumptions of mean and variance homogeneity of the CTD were examined by testing a fully saturated model against a saturated model with equated means and variances within twin pairs and across zygosity for each of the cognitive ability scores. The model with equated means and variances was preferred according to the AIC for all three scales. Based on these results, the means and variances were equated between groups in the full genetic models.

Parameter estimates from the univariate main effects model for cognitive ability test scores are presented in columns 2, 6 and 10 of Table 3. For the total score, additive genetic effects were estimated to account for 29% of the phenotypic variance, shared environmental effects for 47%, and nonshared environmental effects for 19%. PE accounted for the remaining 4% of the phenotypic variance. For the verbal subscore, additive genetic effects accounted for 23% of the phenotypic variance, shared environmental effects for 49%, and nonshared environmental effects for 24% (PE accounted for the remaining 4%). For the nonverbal subscore, 37% of the phenotypic variance were explained by additive genetic, 33% by shared environmental, and 27% by nonshared environmental effects (PE accounted for the remaining 3% of the variance). Shared environmental influences appeared to be stronger for the verbal than the nonverbal subscale.

**Table 3:** Parameter estimates (unstandardized) from main effects model and interaction model for total, verbal, and nonverbal cognitive abilities.

	Total score				Verbal				Nonverbal			
	Main effects		Interaction		Main effects		Interaction		Main effects		Interaction	
Parameter	Estimate	SE	Estimate	SE	Estimate	SE	Estimate	SE	Estimate	SE	Estimate	SE
a	**0.538**	**0.059**	**0.532**	**0.061**	**0.476**	**0.074**	**0.473**	**0.077**	**0.606**	**0.072**	**0.594**	**0.074**
*β*_*x*_			-0.017	0.071			*-0*.*126*	*0*.*078*			-0.006	0.076
c	**0.684**	**0.050**	**0.683**	**0.051**	**0.699**	**0.052**	**0.697**	**0.051**	**0.575**	**0.068**	**0.579**	**0.069**
*β*_*y*_			-0.079	0.056			-0.028	0.046			-0.090	0.071
e	**0.436**	**0.022**	**0.437**	**0.023**	**0.486**	**0.024**	**0.479**	**0.023**	**0.518**	**0.027**	**0.521**	**0.027**
*β*_*z*_			0.017	0.025			**0.049**	**0.021**			0.012	0.029
*β*_*M*_	**0.201**	**0.039**	**0.193**	**0.038**	**0.194**	**0.039**	**0.186**	**0.038**	**0.161**	**0.038**	**0.153**	**0.038**

Parameters in bold are significant at *p* < .05; Parameters in italics are significant at *p* < .10; SE = standard error; a, c, e, = unmoderated elements of genetic, shared, and nonshared path coefficients; a´, c´, e´, = moderated elements of genetic, shared, and nonshared path coefficients.

### G×E analyses

The parameter estimates for the interaction models for the three cognitive ability scores are displayed in columns 3, 7, and 11 of Table 3. Table 4 shows the summary of model fit and model comparisons of the main effects model against the interaction model. The results of the genetic modeling showed no indication of a moderating effect of PE on A, C and E for the total score of cognitive ability. However, a different pattern of results emerged when focusing on the verbal and nonverbal subscales separately. The interaction model for the verbal subscale was the only model that fit the data significantly better than the univariate main effects model. The moderating effect of PE on nonshared environmental effects was significant on a 5% level (*β_z_* = .049; *SE* = 0.021; *p* < .05), implying that nonshared environmental influences on verbal abilities were higher at higher levels of PE. Additionally, there was some, although not statistically significant, indication of a moderating effect of PE on additive genetic effects (*β_x_* = -.126; *SE* = 0.078; *p* < .10), indicating that genetic effects on verbal abilities tended to be higher at lower levels of PE.

**Table 4:** Model fit comparisons.

Scale	Model	-2LL	df	AIC	BIC	diff LL	diff df	*p*
Total score	Interaction	2547.55	1044	459.55	2597.67			
	**Main effects**	**2553.66**	**1047**	**459.66**	**2584.90**	**6.11**	**3**	**0.11**
Verbal	**Interaction**	**2592.02**	**1044**	**504.52**	**2642.64**			
	Main effects	2603.05	1047	509.05	2634.37	10.53	3	0.01
Nonverbal	Interaction	2693.42	1044	605.42	2743.54			
	**Main effects**	**2698.19**	**1047**	**604.19**	**2729.52**	**4.78**	**3**	**0.19**

-2LL = -2 times Log-likelihood of data; df = degrees of freedom; AIC = Akaike’s information criterion; BIC = Bayesian Information Criterion. Best fitting model marked in bold.

**Fig 2:**
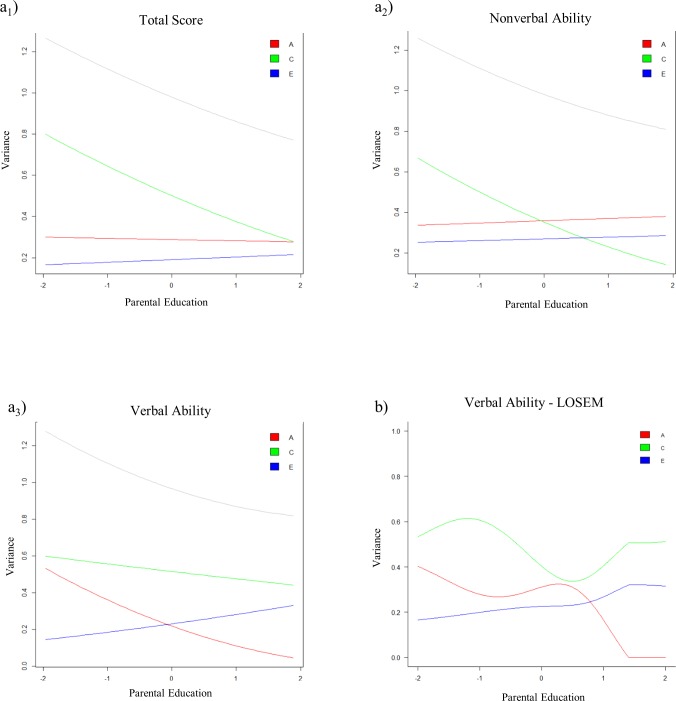
Graphical representation of the G×PE interaction on cognitive abilities. Variance in cognitive-test performance for the three cognitive ability scores accounted for by genetic and environmental factors, graphed as a function of parental education. a) displays the results derived from the Purcell modeling approach; grey line represents the total variance b) displays the results for verbal ability derived from the LOSEM modeling approach. Cognitive test scores were standardized to a z-scale prior to model fitting.

A graphical summary of the moderation analyses, i.e. the variance in cognitive abilities attributable to genetic, shared, and nonshared environmental effects as a function of PE, is given in Fig 2A1 to 2A3. As evident from the figure, the total variance in cognitive ability scores differs across the levels of PE for all three scales, with greater variance at lower levels of PE. The visual summary of the moderation also revealed differences between the verbal and nonverbal subscales. While the nonshared environmental and additive genetic effects for the verbal subscale differed over the range of PE, we found only small differences for the nonverbal subscale. In contrast, the shared environmental effect remained relatively stable for verbal abilities, while for nonverbal abilities, the effect, although not significant, tended to be lower at higher levels of PE.

### LOSEM results for verbal abilities

To evaluate trends in ACE estimates of verbal ability across the range of PE, models were estimated with focal values set between -2 and 2 standard deviations at intervals of 0.01. Results from this model are displayed in Fig 2B. LOSEM findings largely mirrored the results from the continuous model, with two noticeable differences. First, additive genetic influences were high at low levels of PE. They slightly decrease through approximately -1 SD, followed again by a slight increase and leveling off through 0.5 SD and a sharp decline through 1.5 SD. Second, LOSEM results indicated that shared environmental influences on verbal ability first increase through -1 SD followed by a decrease through 0.5 SD and even increase again through approximately 1.5 SD. This is in contrast to the results derived from the continuous model that indicated a nonsignificant linear decline of shared environmental effects over the range of PE. Nonshared environmental influences on verbal ability were linear across PE.

## Discussion

We tested whether genetic and environmental effects on cognitive ability changed as a function of PE in a sample of German twins, aged 7 to 14 years. While our results confirmed a main effect of PE on cognitive ability, we did not find support for a significant effect of SES on the heritability of general cognitive ability, which is in line with recent meta-analytic findings [25]. On a more granular level, however, our results suggest that, for verbal abilities, the nonshared environmental influence varies as a function of PE, in the direction of greater nonshared environmental influences among children raised by more educated parents. It has been hypothesized that the observed cross-national difference of G×SES effects may be driven by cross-national differences in overall socioeconomic inequality, or the degree to which access to high quality education is stratified by social class [24,31].

The Gini index is much higher in the U.S. (e.g., 40.64 in 2010) than in Germany (e.g., 31.14 in 2010; [51]) or Western European countries in general. The Gini index measures the extent to which the distribution of income among individuals or households within an economy deviates from a perfectly equal distribution. A Gini index of 0 represents perfect equality, while an index of 100 implies perfect inequality [51]. The German social security system ensures a primary health and financial care which might imply that the term ‘low SES’ refers to a different poverty threshold compared to the U.S. Moreover, exposure to multiple risks was associated with adverse cognitive development in the U.S. (see [28,52]). Given these differences between nations, more socioculturally diverse genetically informative samples are required to deepen our understanding of the underlying mechanisms via which environments might restrict or foster cognitive development.

Also, access to high quality education might account for differing results between the present German sample and those previously reported for U.S. samples. In Germany, tracking decisions are based on teachers’ recommendations at the end of elementary school based on the overall achievement level of a student [53]. This could potentially enable bright, but underprivileged, children a greater access to rigorous educational tracks.

Finally, intellectually stimulating proximal environments have been assumed to form a stable environmental basis upon which “genetic potentials for *effective* psychological functioning are actualized” [11]. However, it is possible that intellectual stimulations, or proximal processes (that children are exposed to), depend less on parental education in the present sample. Or, put differently, lower parental education might simply not be an adequate indicator of an intellectually less supportive rearing environment in the population sampled here. As proximal processes are assumed to not only be provided by parents, but, especially as children get older, also by teachers and other caretakers [11], schools may provide a second source for proximal processes. This is compatible with studies reporting that the minimal standard of school quality, as well as school quality by neighborhood of residency, may be different in Germany compared to the U.S. (e.g., [54; 55]).

With regard to the results of our study, one might also hypothesize that verbal test performance is more closely related to educational processes and social background than nonverbal test performance, as individual differences in verbal abilities at the upper end of the PE distribution are to a greater extent explained by processes that contribute to differences between family members (i.e., nonshared environmental factors). Compared to the nonverbal test, the verbal test might therefore be more influenced by PE. With regard to our results this would imply that verbally gifted children at the lower end of the PE distribution would strive for suited environments, and would therefore benefit the most from a supporting school environment. This pattern has become more differentiated when applying the more fine-grained LOSEM method. Here, our results suggested that the trend across levels of PE was not strictly linear. Although being comparable at the extreme upper and lower end of the distribution, effects of the shared environment appeared to be different for families in the middle of the PE distribution. This may be one reason why shared environmental effects were not significant in the classic Purcell model.

The present study also bears several limitations. First, SES was measured at the family level, which prevents us from decomposing its variance into genetic and environmental components. Variables measured at the family-level enter into twin models as indices of the shared environment. Because the main effect of SES was controlled in our models, the estimates of shared environmental variance represent shared environmental variance above and beyond parental education [56]. Moreover, because SES cannot be decomposed into genetic and environmental variance components, we are unable to explicitly test for a genetic correlation of the moderator and the trait [57]. It should also be noted, that available statistical models of G×E are limited in their ability to adequately investigate potentially underlying processes of what is observed as changing heritability as a function of an environment variable. Here, genetically sensitive, large scale longitudinal studies that measure multiple, changing, aspects of children’s proximal experiences and broader contexts would be valuable for specifically investigate the role of rGE in the development of cognitive abilities. Our study would have benefited from employing further indicators of parental SES, and measures of the home environment (e.g., parenting style, parental involvement, family stressors, or chaos in the home), that might better reflect relevant factors for the quality of proximal processes on the family level (see e.g., [30]). Incorporating such measures into future data collection efforts should be considered a major priority.

Finally, although our sample size is comparable to those of many other twin studies [41], it is still at the lower end and much smaller than most epidemiological studies. Replication is key, and we encourage researchers to continue to report tests of G×SES interactions on cognitive abilities, particularly using large, well-powered, samples that cover the full range of SES [5]. Future research with information on genetic markers should also test the differential susceptibility hypothesis that assumes non-linear relation of moderator and phenotype.

In conclusion, our results illustrate the importance of environmental circumstances in general and family background in particular for the genesis and explanation of interindividual differences in verbal test performance and ability. We found evidence that G×PE interactions might not play out for the full range of cognitive abilities but for those abilities that are more closely related to SES markers, such as verbal ability [2].
